# Memory characteristics of silicon nanowire transistors generated by weak impact ionization

**DOI:** 10.1038/s41598-017-12347-x

**Published:** 2017-09-29

**Authors:** Doohyeok Lim, Minsuk Kim, Yoonjoong Kim, Sangsig Kim

**Affiliations:** Department of Electrical Engineering, Korea University, 145 Anam-ro, Seongbuk-gu, Seoul, 02841 Republic of Korea

## Abstract

In this study, we demonstrate the static random access memory (SRAM) characteristics generated by weak impact ionization in bendable field-effect transistors (FETs) with n^+^-p-n^+^ silicon nanowire (SiNW) channels. Our bendable SiNW FETs show not only superior switching characteristics such as an on/off current ratio of ~10^5^ and steep subthreshold swing (~5 mV/dec) but also reliable SRAM characteristics. The SRAM characteristics originate from the positive feedback loops in the SiNW FETs generated by weak impact ionization. This paper describes in detail the operating mechanism of our device and demonstrates the potential of bendable SiNW FETs for future SRAM applications.

## Introduction

Electronic devices fabricated on lightweight, bendable, transparent, and low cost plastic substrates are believed to have great potential application in future wearable electronics. Bendable static random access memories (SRAMs) using organic semiconductors^1^ and ultrathin body silicon on insulator^2^ have been intensively explored for wearable electronic systems today owing to their fast data-access time. However, the conventional SRAM structure, which is composed of at least six transistors, requires a large area, although the SRAM density and performance are usually enhanced by scaling down the device dimensions^3^. In addition, the leakage power of a SRAM, which is typically constructed from complementary metal-oxide semiconductor (CMOS) technology, is the main component of the power consumption; all issues associated with MOS field-effect transistor (MOSFET) scaling apply to the scaling of SRAM. In recent years, power dissipation has become a major problem because of the fundamental limits brought by the continuous downscaling of MOSFETs^4,5^. The 60-mV/dec subthreshold swing (SS) at room temperature is the theoretical limit of conventional MOSFETs due to the physical limitation from the charge diffusion mechanism and Boltzmann statistics, thus preventing further reductions in leakage power. Therefore, new mechanisms should be explored to go beyond this limitation and realize future wearable electronics.

Various promising devices that realize steep slopes beyond that of the MOSFET technology have been introduced, including tunneling FETs (TFETs)^6–9^, impact ionization FETs^10–12^, and feedback FETs (FBFETs)^13,14^. Although these devices that operate in innovative operating mechanisms such as band-to-band tunneling (BTBT), avalanche breakdown, and positive feedback loop show low SS values, they suffer from some major drawbacks in their device application. For the TFETs, low on-current remains a common issue in spite of the many techniques to improve their drivability. For the impact-ionization FETs, severe hot carrier effects lead to significant threshold voltage shifts due to the injected carriers into the gate dielectric. Compared with other steep switching devices, FBFETs are the most promising candidate for future switching devices. The FBFETs operate with potential barrier modulation for electrons and holes using p^+^-i-n^+^ diodes and gate sidewall spacers. For the FBFETs, however, high-voltage operation and complicated programming for charge trapping are required, which may cause unintentional degradation or failure^15^. Although alternative device structures without any trapped charges have been introduced, it is inevitable that a dual gate voltage modulation of a four-terminal device structure generates a positive feedback loop for steep switching behavior^16–19^. On the other hand, Z. Lu *et al*. proposed a new type of positive feedback loop, which is generated by the floating-body effect of silicon-on-insulator (SOI) transistors and weak impact ionization^20^. The basic structure of FET is similar to a fully depleted SOI MOSFET that uses the standard CMOS technology; thus, this device has a three-terminal device structure similar to that of the MOSFETs. Nevertheless, both FETs that operate by positive feedback loops suffer from hysteresis characteristics, which means that the turn-on and turn-off of the devices occur at different gate bias voltages^15–20^.

Recently, FBFETs with p^+^-i-n^+^ diodes have been successfully utilized for dynamic random access memory (DRAM) without any external capacitor owing to their sharp switching and hysteresis characteristics^21–23^. Moreover, a thyristor-based RAM and a field-effect diode with two front gates, which are operated by a similar positive feedback loop, have been proposed for SRAM applications^24–28^. For the positive feedback loop generated by weak impact ionization, capacitorless DRAM functionalities have been reported in recent years^29,30^. In spite of the superior DRAM functionalities, the data-retention time is limited because there is no neutral region of the body in fully depleted silicon-on-insulator (FD SOI); thus, back-gate biasing is required to accommodate the majority carriers in the accumulated back channel. This makes the implementation of SRAMs difficult. In this regard, three-dimensional (3-D) gated structures realized using partially depleted silicon nanowires (PD SiNWs), which provide neutral region for storage of carriers and excellent electrostatic control of the channels, can possibly overcome retention failure. In addition, the use of an SOI substrate is an obstacle to achieve bendable electronics. Based on the insights into the floating body of PD SiNW, we experimentally confirm SRAM characteristics generated by weak impact ionization in a newly designed n^+^-p-n^+^ PD SiNW FET on bendable substrates. The SiNW used in this study is fabricated by top-down route that is fully compatible with the current CMOS technology. Furthermore, we demonstrate the feasibility of full SRAM operations in our PD SiNW FET which is connected in series with an access transistor by computer simulation tool.

## Results and Discussion

**Figure 1:**
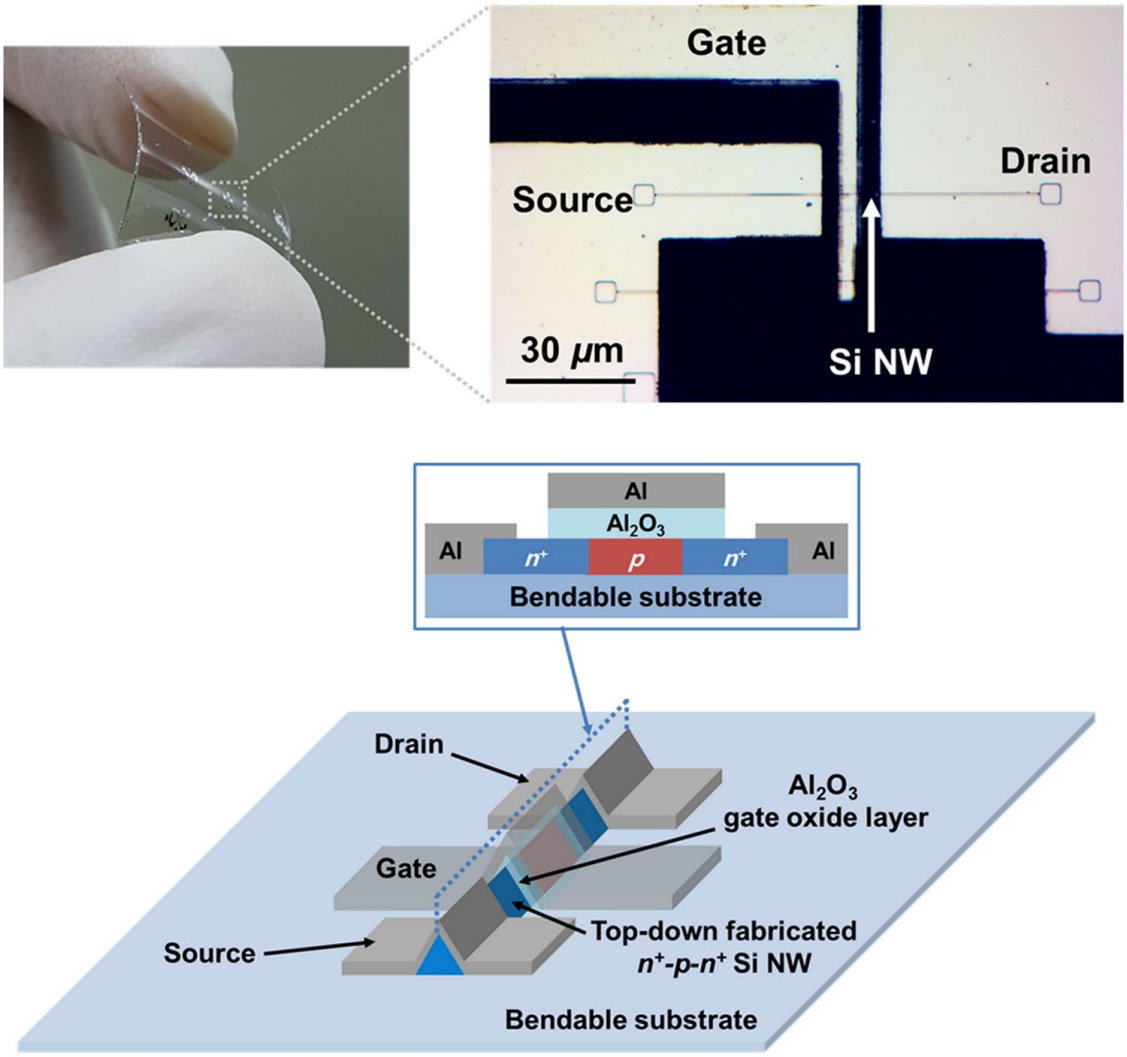
Optical images and schematic illustration of the bendable n^+^-p-n^+^ SiNW FET.

The optical images and schematic device illustration of an n^+^-p-n^+^ SiNW FET on a bendable substrate are shown in Fig. 1. A top-down-fabricated n^+^-p-n^+^ doped SiNW is used as an active channel with a p-region channel length of 700 nm. The SiNW has a triangular shape with a width and height of ~150 nm. At the upper side of the channel region, a high-*k* Al_2_O_3_ gate oxide layer and a gate electrode are stacked. Compared with a planar structure, the use of SiNW for the FET enables a 3-D gated structure, which leads to an increase in the gate coverage. Therefore, we expect that the SiNW FET can provide superior electrical characteristics to the planar structure-based FETs. These unique operation mechanisms are presented in more details in the following section.

**Figure 2:**
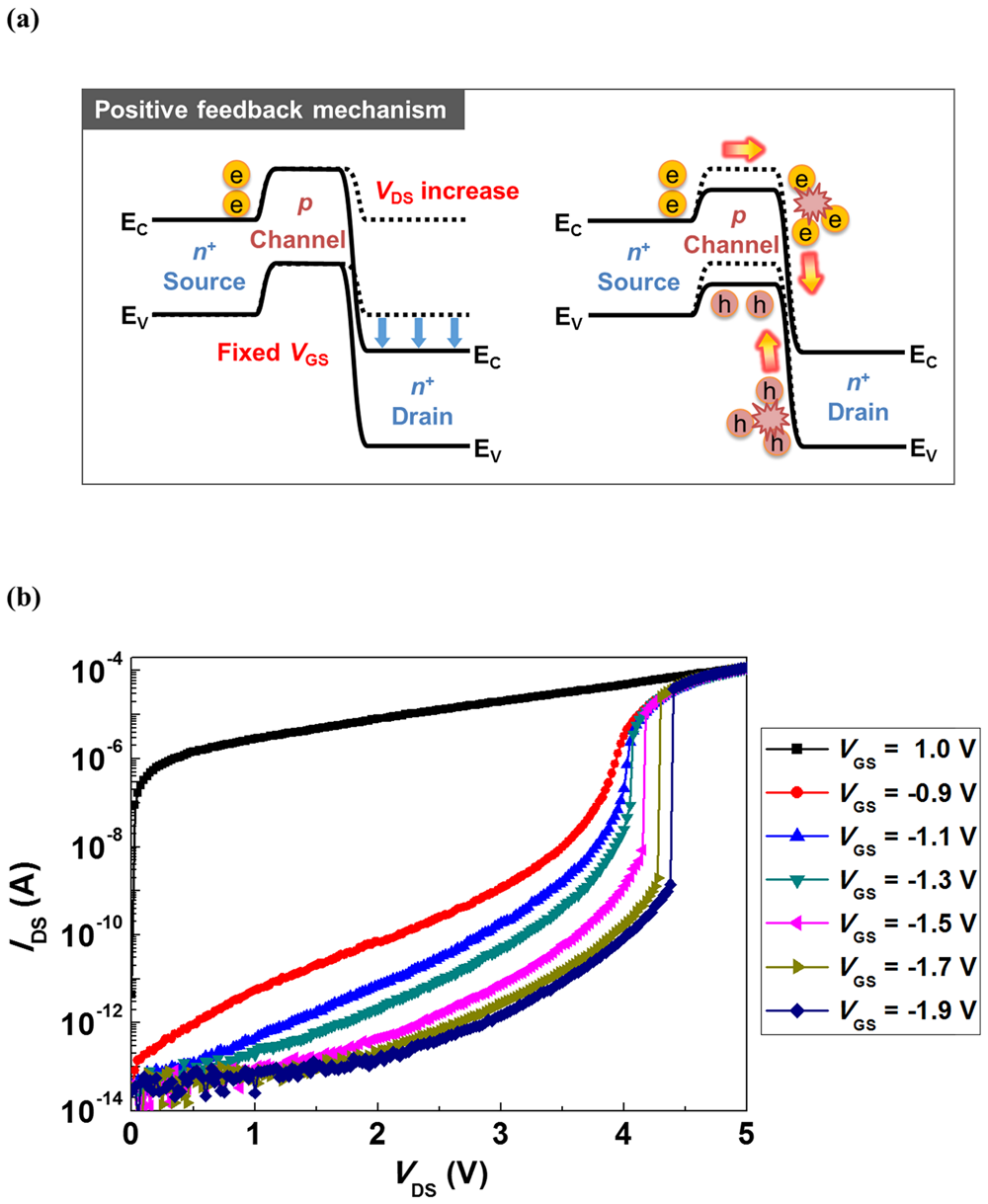
(**a**) Energy band diagrams of the positive feedback mechanism. (**b**) Output characteristics of the n^+^-p-n^+^ SiNW FET.

The energy band diagrams of the positive feedback mechanism induced by weak impact ionization of the n^+^-p-n^+^ SiNW FET are shown in Fig. 2(a). Weak impact ionization is defined as the impact ionization, which only occurs during the transition and vanishes when the device completely turns on [see Supplementary Information]. When *V*_DS_ reaches the point that triggers weak impact ionization near the drain junction, the weak impact ionization generates electron/hole pairs. The generated electrons flow into the drain, and the holes accumulate in the body of the SiNW, thereby increasing the body potential. An increase in the body potential, *i.e*., reduction in the threshold voltage in the MOSFET, induces creation of more holes by the weak impact ionization. More created holes injected into the SiNW body result in further increase in the body potential, which leads to an increase in *I*_DS_ and more impact ionization current. Thus, a positive feedback loop is formed. As result, the weak impact ionization triggers a positive feedback loop.

The drain current (*I*_DS_) versus drain-to-source voltage (*V*_DS_) characteristics of the n^+^-p-n^+^ SiNW FET is shown in Fig. 2(b). When positive gate bias voltages are applied to the transistor, the *I*_DS_–*V*_DS_ characteristics behave according to a traditional output curve of the conventional MOSFET (*e.g*., *V*_GS_ = 1 V). However, we note that abnormal *I*_DS_ characteristics are observed in Fig. 2(b) when negative gate bias voltages (*e.g*., *V*_GS_ = −0.9 V to −1.9 V) are applied, *i.e*., in the subthreshold region. As *V*_DS_ is forwardly swept from 0 to 5 V, *I*_DS_ abruptly increases at certain points due to the positive feedback mechanism. Figure 2(b) shows that the latch width, defined as the width of the abrupt change in the drain current (*I*_DS_), can be determined by the gain of the positive feedback loop. A higher gain of the positive feedback loop induces a wider latch width. The weak impact ionization is a dominant factor in the gain of the positive feedback loop when the weak impact ionization triggers a positive feedback loop. Under a relatively high negative gate bias voltage, holes are accumulated at the SiNW surface; hence, it behaves similar to a p-region that is more heavily doped. Thus, the depletion region of the p-n^+^ junction at the SiNW surface becomes narrower, causing an increase in the local electric field. Although the local electric field increases, a higher lateral electric field, *i.e*., a higher drain voltage, is required for the weak impact ionization because the depletion region becomes narrower. Weak impact ionization can occur when the kinetic energy of the accelerated carriers along the depletion region is greater than the band-gap energy before colliding with atoms in the lattice. Moreover, the carriers accelerate and gain sufficient energy due to the higher drain voltage, which leads to an increase in the weak impact ionization rate. Therefore, a higher drain voltage is required to trigger a positive feedback loop induced by the weak impact ionization as the gate bias voltage is more negatively applied (*V*_DS_ = 4.375 V for *V*_GS_ = −1.9 V, *V*_DS_ = 4.275 V for *V*_GS_ = −1.7 V, *V*_DS_ = 4.15 V for *V*_GS_ = −1.5 V, and *V*_DS_ = 4.05 V for *V*_GS_ = −1.3 V). In addition, the gain of the positive feedback loop becomes higher, *i.e*., the latch width becomes wider, because a more negative gate bias voltage leads to an increase in the weak impact ionization rate. As fixed *V*_GS_ changes from −1.9 to −0.9 V, the latch width reduces and disappears due to the reduction in the gain of the positive feedback loop induced by the weak impact ionization.

**Figure 3:**
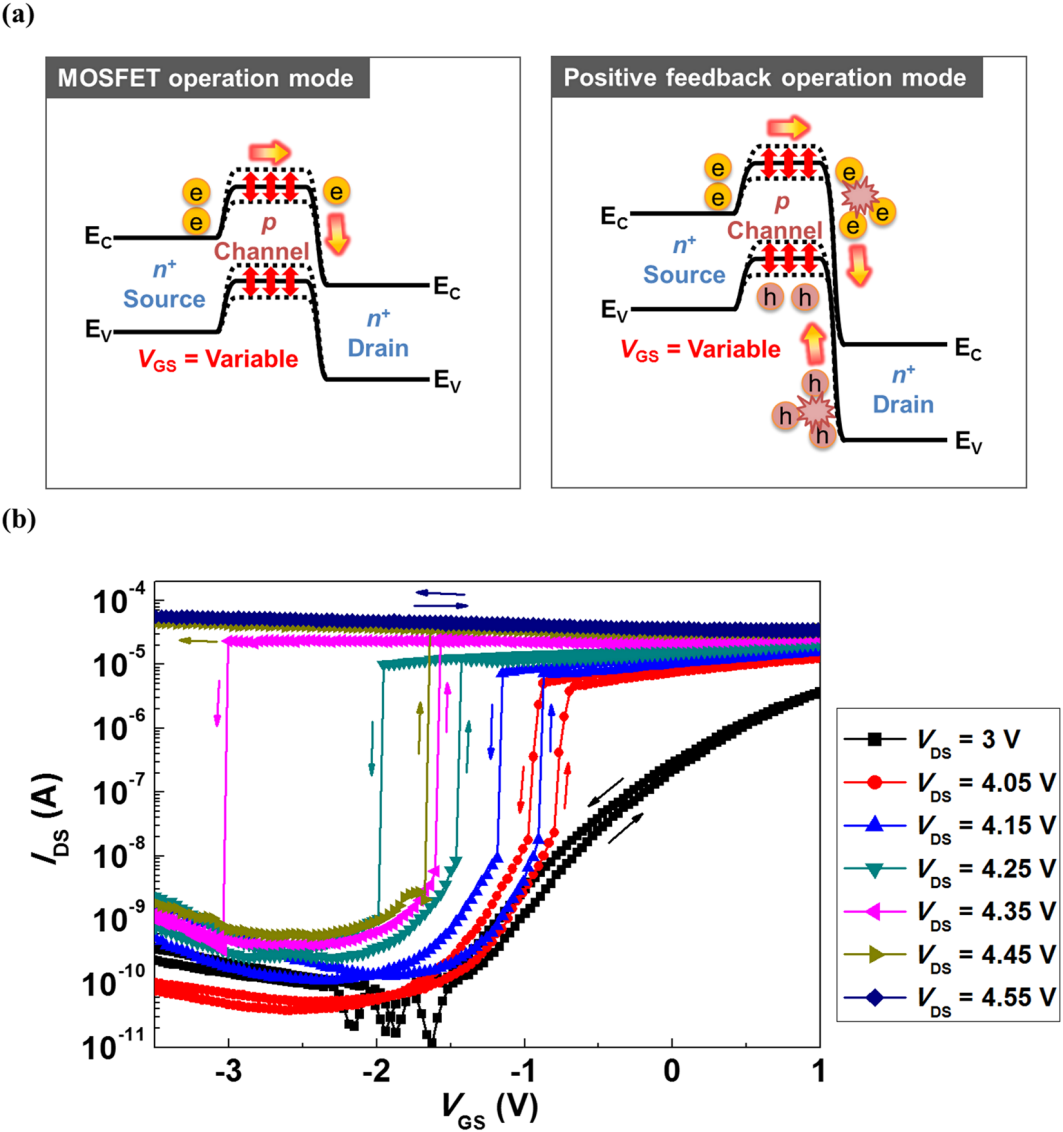
(**a**) Energy band diagrams of the n^+^-p-n^+^ SiNW FET in the MOSFET and positive feedback operating modes. (**b**) Transfer curves of the n^+^-p-n^+^ SiNW FET with various *V*_DS_.

Next, we will analyze the latch phenomenon using the positive feedback loop in the *I*_DS_ versus *V*_GS_ characteristics of our n^+^-p-n^+^ SiNW FET. Figure 3(a) shows the energy band diagrams of the SiNW FET in the MOSFET and the positive feedback operating modes. The operating mode of the SiNW FET can be controlled by modulating *V*_DS_. To set the MOSFET operating mode, *V*_DS_ is fixed to a relatively low bias, and *V*_GS_ is varied. However, the positive feedback operating mode can be set by fixing a relatively high *V*_DS_ and varying *V*_GS_. Under relatively high *V*_DS_, weak impact ionization triggers a positive feedback loop when the gate voltage is swept. Figure 3(b) shows the *I*_DS_–*V*_GS_ transfer curves of the SiNW FET with various *V*_DS_. At a relatively low drain voltage (*e.g*., *V*_DS_ = 3 V), our SiNW FET shows a conventional MOSFET *I*_DS_–*V*_GS_ transfer curve. However, novel characteristics are observed in our SiNW FET under a relatively high drain voltage bias (*e.g*., *V*_DS_ > 4 V); *I*_DS_ abruptly increases/deceases by 3–5 decades as *V*_GS_ is swept upward/downward. The abrupt increase/decrease in the drain current corresponds to the “latch-up/down” phenomena, which have also been observed in biristors^31,32^, thyristors^33^, and field-effect diodes^34^. In our SiNW FET, the positive feedback loop triggers the latch-up/down phenomena by sweeping the gate voltage. When the gate voltage is swept upward, the device exhibits a latch-up phenomenon triggered by the positive feedback loop. When a relatively high negative *V*_GS_ is applied, the hole concentration at the SiNW surface is high, and the width of the depletion region in the p-n^+^ junction is very narrow. When the gate bias voltage is less negatively applied, the width of the depletion region increases due to the reduction in the hole accumulation at the SiNW surface. Thus, the accelerated charge carriers due to the drain voltage have sufficient kinetic energy owing to the wider width of the depletion region, which leads to the weak impact ionization at the drain junction, thereby very quickly generating a positive feedback loop. As a result of the positive feedback loop, a latch-up phenomenon is observed at the latch-up voltage (*V*_latch-up_), defined as the gate voltage when the positive feedback loop induced by the weak impact ionization triggers the latch-up phenomenon. In addition, a latch-down phenomenon is observed in the device when the gate voltage is swept downward. The positive feedback loop is eliminated when the gate bias voltage is more negatively applied; the more negatively applied *V*_GS_ induces high hole concentration at the SiNW surface, which leads to a narrow depletion region in the p-n^+^ junction and thereby suppressing the weak impact ionization. The latch-down phenomenon is observed at the latch-down voltage (*V*_latch-down_), defined as the gate voltage when the positive feedback loop is eliminated by suppressing the weak impact ionization.

The latch-up/down phenomena exhibit different characteristics under variations in the fixed drain biases. First, the widths of the latch-up/down phenomena increase as fixed drain bias *V*_DS_ vary from 4.05 to 4.45 V in 1-V steps except for the latch-down phenomenon at *V*_DS_ = 4.45 V. The widths of the latch-up/down phenomena can be evaluated using the point SS (SS_point_) values, defined as the inverse slope of the logarithmic *I*_DS_ versus gate voltage characteristic, which is expressed by the equation

1 $${[{\rm{d}}({\mathrm{log}}_{10}(|{I}_{{\rm{DS}}}|)/{\rm{d}}{V}_{{\rm{GS}}})]}^{-1}$$

The wide widths of the latch-up/down phenomena imply that the SS_point_ values are small. As fixed *V*_DS_ changes from 4.05 to 4.35 V in 1-V steps, SS_point_ decreases (11.3 → 9.5 → 7.8 → 6.8 mV/dec for the latch-up phenomenon and 19.1 → 8.6 → 6.2 → 5.1 mV/dec for the latch-down phenomenon). The changes in the widths of the latch-up/down phenomena imply that *V*_DS_ plays an important role in the gain of the positive feedback. A higher impact ionization rate at larger *V*_DS_ enables a higher gain positive feedback loop. In particular, a larger *V*_DS_ (reverse bias in the p-n^+^ junction) value widens the depletion region and accelerates the charge carriers in the depletion region, thereby increasing the impact ionization rate. However, the device does not show that the latch-down phenomenon at *V*_DS_ is fixed at 4.45 V, whereas the latch-up phenomenon is observed at SS_point_ = 5.8 mV/dec. This result indicates that the device remains in the latch-up state as long as *V*_DS_ is applied when the positive feedback loop gain is sufficiently high. When *V*_DS_ reaches 4.55 V, the device does not show the latch-up/down phenomena. The device normally turns on, which implies that the latch-up/down phenomena can be created under appropriate *V*_DS_ values, enabling the generation of the positive feedback loop induced by weak impact ionization; a more detailed latch-up/down phenomena with weak impact ionization in our SiNW FET is described in the Supplementary Information.

In addition, hysteresis windows, defined as the difference between *V*_latch-up_ and *V*_latch-down_, are observed. As fixed drain bias *V*_DS_ varies from 4.05 to 4.35 V in 1-V steps, the device exhibits a shift in *V*_latch-up_ (−0.8 → −0.905 → −1.465 → −1.605 V) and *V*_latch-down_ (−0.94 → −1.15 → −1.955 → −3.005 V). The shift in *V*_latch-up_ is determined by fixed *V*_DS_. When fixed *V*_DS_ is 4.05 V, a wide depletion region in the p-n^+^ junction at the SiNW surface is required to initiate a weak impact ionization. The gate is somewhat negatively biased (*V*_latch-up_ = −0.8 V) to widen the depletion region, thereby triggering a positive feedback loop induced by the weak impact ionization. At *V*_DS_ = 4.35 V, however, *V*_latch-up_ is more negative (*V*_latch-up_ = −1.605 V) because the impact ionization rate is sufficiently high to generate a positive feedback loop. The lateral electric field (*V*_DS_) is the dominant factor in the weak impact ionization, although the width of the depletion region induced by the more negatively biased gate voltage is too narrow to initiate weak impact ionization. Consequently, *V*_latch-up_ shifts in the negative direction as *V*_DS_ increases. In addition, a larger *V*_DS_ value leads to a higher impact ionization rate, thereby generating a higher gain positive feedback loop. In particular, more generated holes are accumulated in the SiNW channel region by the high impact ionization rate, which helps the impact ionization to be further activated. The latch-down state requires more negative *V*_GS_ to suppress the latch-up state as *V*_DS_ increases. We note that *V*_latch-down_ shifts to a more negative direction than *V*_latch-up_ due to the increase in the holes that are accumulated in the Si NW channel region as *V*_DS_ increases. For these reasons, a higher drain bias voltage causes wider hysteresis windows in the counterclockwise direction. These hysteric characteristics imply that the n^+^-p-n^+^ Si NW FET can be used as a memory device.

**Figure 4:**
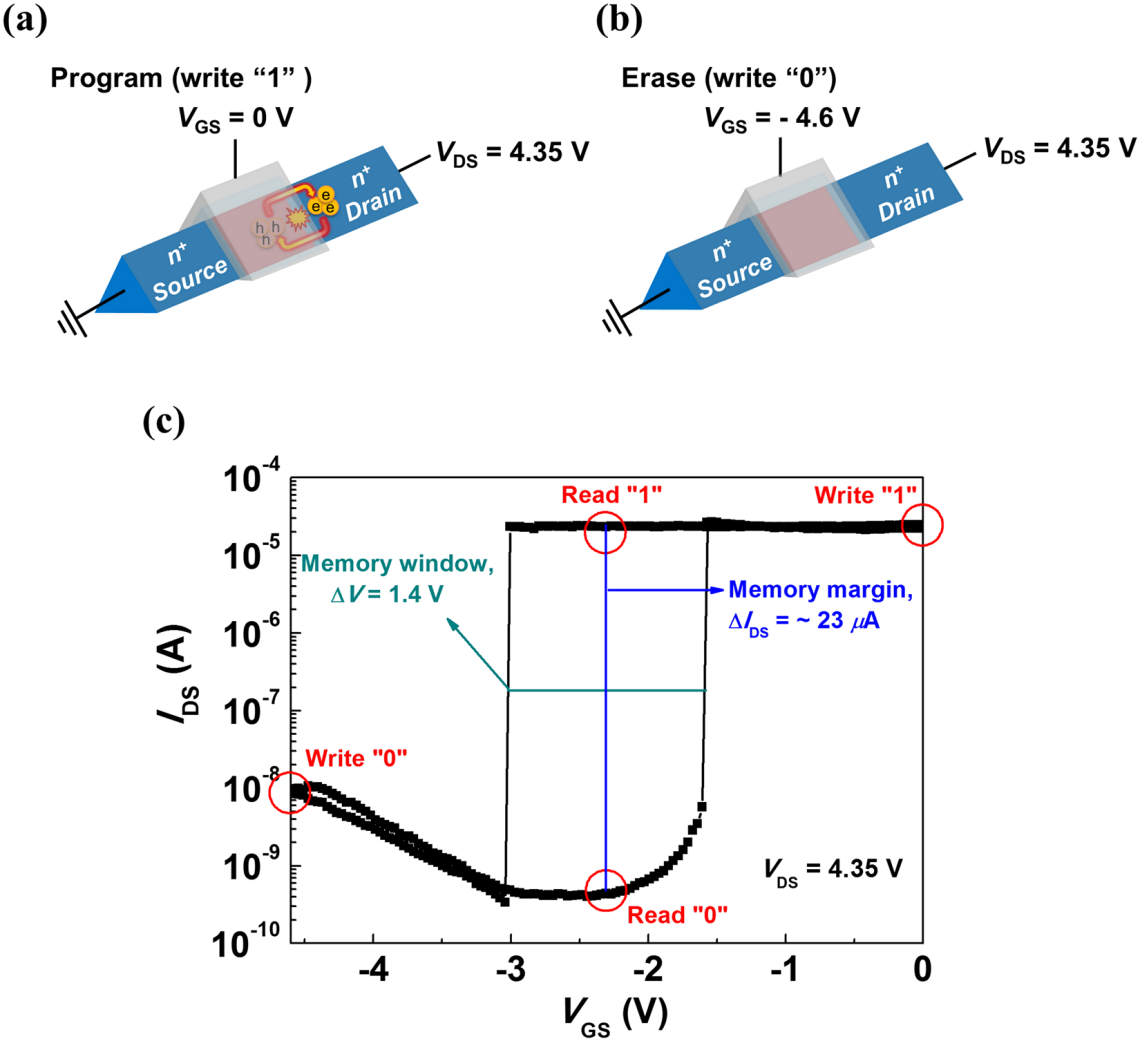
Schematic illustration for (**a**) program (write “1”) and (**b**) erase (write “0”). (**c**) Memory characteristics of the n^+^-p-n^+^ SiNW FET based on hysteresis characteristics of the *V*_GS_–*I*_DS_ curve.

Based on the operating principle of the positive feedback loop by weak impact ionization, we then investigated the feasibility of our device for memory applications. Figure 4 shows the operating conditions and performance parameters of the n^+^-p-n^+^ SiNW FET based on the *I*_DS_–*V*_GS_ hysteresis curve at *V*_DS_ = 4.35 V. The SiNW FET can utilize the narrow memory window due to the high memory margin caused by the excellent SS characteristics. To write “1” data into the SiNW FET, *V*_GS_ is biased at 0 V and *V*_DS_ is fixed at a bias of 4.35 V to reach the latch-up state [Fig. 4(a)]. On the other hand, to write “0” data into the SiNW FET, *V*_GS_ is triggered to −4.6 V and *V*_DS_ is fixed at a bias of 4.35 V to suppress the positive feedback loop, thereby reaching the latch-down state [Fig. 4(b)]. The read operation is performed by sensing the difference in the drain current between the “1” and “0” states. Note that the SiNW FET data between the “1” and “0” states can be distinguished from each other by sensing the difference in the read current at *V*_GS_ = −2.3 V and *V*_DS_ = 4.35 V. The differences in the read current and latch-up/down voltage between the two data states, which are indexes of the memory margin and memory window, are measured as ~23 *μ*A and 1.4 V, respectively. The summary of the operating conditions of the SiNW FET for evaluation of the SRAM characteristics is listed in Table 1.

**Table 1:** Operating conditions of the n^+^-p-n^+^ Si NW FET for the evaluation of the SRAM characteristics.

	Program (write “1”)	Erase (write “0”)	Read
*V*_*GS*_ (V)	0	–4.6	–2.3
*V*_*DS*_ (V)	4.35	4.35	4.35

**Figure 5:**
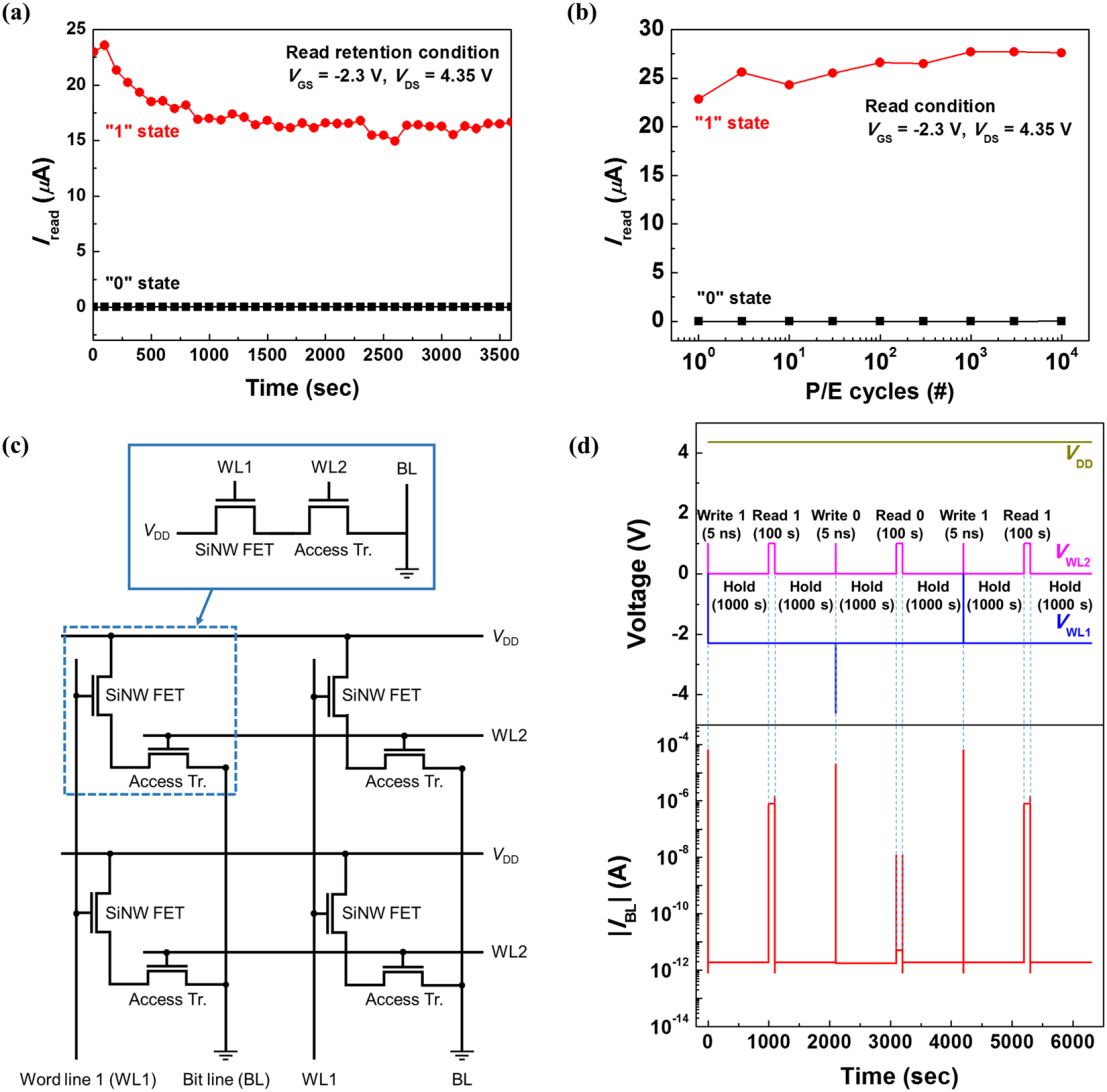
(**a**) Read retention characteristics at *V*_GS_ = −2.3 V and *V*_DS_ = 4.35 V. (**b**) Endurance characteristics as a function of the number of P/E cycles. (**c**) 2 × 2 SRAM cell array structure consisting of a SiNW FET and an access transistor. (**d**) Simulated timing diagrams of an SRAM array cell operation.

In order for the n^+^-p-n^+^ Si NW FET to investigate the SRAM characteristics, its retention and endurance characteristics should be examined. Figure 5(a) shows the read retention characteristics measured at room temperature under the read conditions of *V*_GS_ = − 2.3 V and *V*_DS_ = 4.35 V. Before measuring the data retention, programming and erasing operations are performed. Although pulse generators can offer a pulse width of a few nano seconds, they cannot generate the constant voltage because the total amount of the sampling is limited. Accordingly, a pulse width of 500 *μ*s is utilized to measure the long retention time using the sampling measurements in the semiconductor parameter analyzer (Agilent 4155 C). The “1” state is programmed by generating a positive feedback loop with *V*_GS_ = 0 V and *V*_DS_ = 4.35 V for 500 *μ*s. Although the read current (*I*_read_), which corresponds to *I*_DS_ under the read condition, reaches values of less than ~23 *μ*A after 122 s, the “1” state reading is well maintained for 3600 s. At the read voltage, recombination of the excess holes, which are accumulated in the SiNW body by the positive feedback loop at the programming voltage, reduces the body potential, reducing the positive feedback gain and leading to a slight decrease in the current level of the “1” state reading. However, the “1” state reading still triggers a positive feedback loop, which induces the latch-up state. This result implies that the “1” state reading enables a nondestructive read operation as well as stable data-retention characteristics. Moreover, the “0” state is set by eliminating the positive feedback loop with *V*_GS_ = −4.6 V and *V*_DS_ = 4.35 V for 500 *μ*s. The “0” state reading shows stable *I*_read_ characteristics at “0” state for 3600 s. Although *I*_read_ for the “0” state slightly increases due to the gate-induced drain leakage (GIDL) caused by BTBT, it remains between ~10^–9^ and ~10^–8^ A. In particular, the GIDL generates excess holes in the SiNW channel body, which increases the body potential of the SiNW and leads to a slight increase in *I*_read_ in the “0” state. Triangular geometry has been reported to minimize the GIDL, which induces the hole generation^35^. Our triangular-shaped SiNW FET does not show a “0” state reading failure for 3600 s because the generated holes by BTBT are not sufficient to trigger a positive feedback loop. As a result, the memory margin remains over 15 *μ*A for 3600 s, which implies that the degradation of the “1” and “0” states is negligible. Figure 5(b) shows the endurance characteristics as a function of the number of programming/erase (P/E) cycles. During the endurance examination, a pulse width of 500 *μ*s was applied for programming and erasing. Although the “1” state read current slightly increases due to the iterative and fast positive feedback loop generation, the memeroy margin shows reliable characteristics even after 10^4^-cycle PE operations. To further investigate full SRAM operations, a computer simulation is performed using device simulator (Silvaco Atlas, version 5.20.2.R)^36^. In this simulation, an n-channel MOS (NMOS) access transistor is connected in series with our SiNW FET. Figure 5 (c) shows 2 × 2 SRAM cell array structure consisting of a SiNW FET and an access transistor. A wordline 1 (WL1) controls the binary information and is connected to the gate of the SiNW FET. The power supply (*V*_DD_) should be applied for sustaining the state of the positive feedback loop. The access transistor acts as a switch and is addressed by a wordline 2 (WL2). Figure 5 (d) shows simulated timing diagrams of an SRAM cell operation. Our SRAM array cell provides the fast writing speed and the nondestructive reading. The positive feedback loop is not affected by the hold operation, which corresponds to static retention characteristics. Moreover, the storage data in a half-selected SRAM cell does not show the failure when WL1 signal is enabled. The simulation results including the timing diagrams of the half-selected SRAM cell are summarzied in more details in the Supplementary Information.

Table 2 shows the comparison between the memory characteristics of our SRAM array cell and the conventional 6T-SRAM. In the current CMOS technology, the standby power is one of the most important issues because it is about a third of the total power. Although the operation voltage (*V*_DD_) in our SRAM array cell is large, the standby power is low due to small leakage current from a very few number of transistors at the standby state. A further channel length scaling down in our SiNW FET could lead to the reduction in *V*_DD_. In addition, the operation time is 5 ns, which is comparable to the conventional six transistor-SRAM (~ 1 ns). With regard to the cell area, a small size of our SRAM array cell (8 F^2^) can overcome the limit to the cell area reduction in the conventional 6T-SRAM (140 F^2^)^37^.

**Table 2:** Comparison of the memory characteristics of SRAM array cell.

	**Device type**	**Operation time**	**Area size**	**Standby power**	***V***_**DD**_
Conventional	Standard 6T–SRAM	~1 ns	140 F^2^	~1 nW	<1 V
This work	SiNW FET SRAM array cell	5 ns	8 F^2^	~10 pW	4.35 V

(Feature size = F).

**Figure 6:**
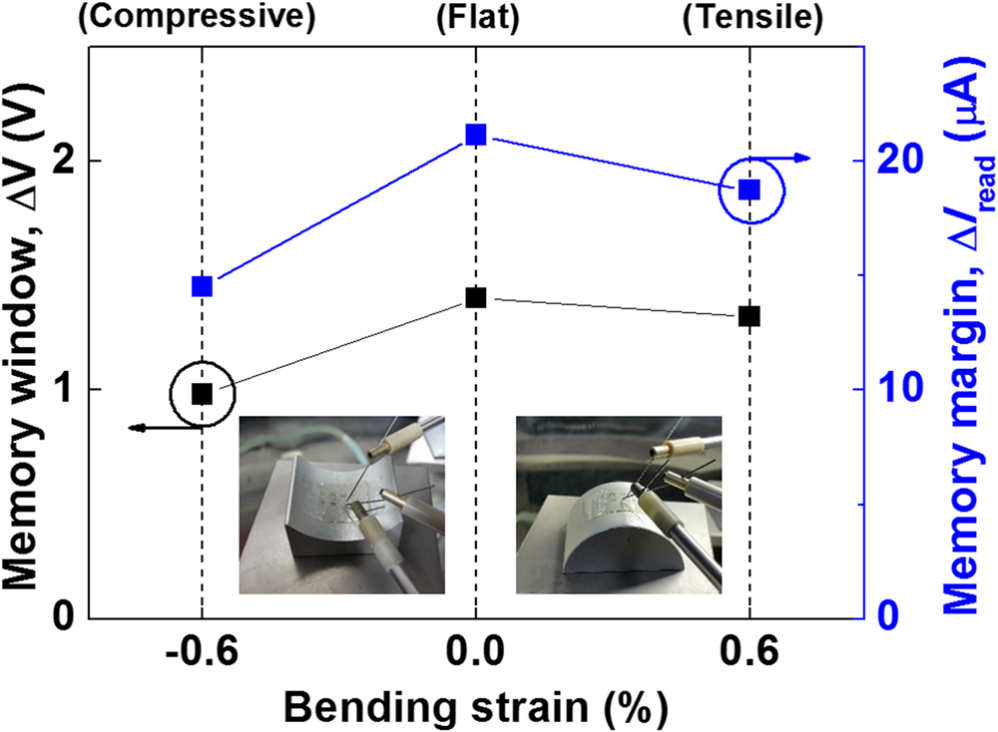
Variation in the memory characteristics as a function of the bending state. The inset shows optical images of the devices under (left) compressive and (right) tensile stress in the bending stages.

To evaluate the mechanical bendability of our n^+^-p-n^+^ SiNW FET, the memory characteristics are investigated under various bent states. The task is performed by bending the plastic substrate in the channel transport direction to apply tensile or compressive strain. The applied strain value is 0.6%; the strain value is obtained from the following expression:

2 $$Strain\,( \% )=100\times \frac{{t}_{substrate}+{r}_{SiNW}}{2\times {R}_{c}}\,$$

where the substrate thickness (t_substrate_) is 200 *μ*m, the SiNW radius (r_SiNW_) is 150 nm, and the radius of curvature (R_c_) is 18 mm^38^. Figure 6 shows the variation in the memory characteristics of the SiNW FET in the flat- and bent-substrate states. Compared with the flat state, the memory characteristics (including the memory window and memory margin) under tensile and compressive strain are slightly changed. The applied strains affect the physical properties of the SiNW and the gate oxide layer when the device is in the upward and downward bent states. The strains cause band splitting in the SiNW^39^ and generation of the traps located at the gate oxide layer. This physical degradation under the bent states suppresses the floating-body effect of the SiNW FET^40^, thereby decreasing the positive feedback loop gain. Therefore, the decrease in the positive feedback loop gain caused by the strains leads to slight degradation in the memory characteristics, including the memory window and memory margin, as shown in Fig. 6. In spite of the compressive and tensile strains, our SiNW FET shows reasonable memory characteristics.

## Conclusions

We have demonstrated a bendable FET with SRAM characteristics generated by weak impact ionization in a fully CMOS-compatible n^+^-p-n^+^ SiNW channel. Using a positive feedback loop, our n^+^-p-n^+^ Si NW FET exhibits superior switching characteristics, including ~5 mV/dec SS with ~10^5^ *I*_on_/*I*_off_ at *V*_DS_ = 4.35 V. Furthermore, the device shows SRAM characteristics with 3600 s of retention time, 10^4^ PE cycles of endurance, and high memory margin of more than 15 *μ*A. Considering that the conventional SRAM offers a read current of less than 10 *μ*A, our SiNW FET can identify the data state without a sense amplifier. Moreover, our simulation work clarify the full SRAM operations. Therefore, these results show that our SiNW FET opens up a possible alternative to the conventional six-transistor SRAM.

## Methods

### Device Fabrication

First, n^+^-p-n^+^ SiNWs were derived from a (100)-orientation bulk Si wafer (doping concentration = ~10^16^ atoms/cm^3^) using a CMOS-compatible top-down route, *i.e*., photolithography combined with anisotropic wet etching using a tetramethylammonium hydroxide solution, thermal oxidation, and ion implantation doping. The doping concentrations of p for the channel region and n^+^ for the drain/source regions are ~10^17^ and ~10^20^ cm^−3^, respectively. The SiNWs were transferred onto a bendable polyethersulfone substrate through the direct transfer method^41^. Al source, drain, and gate electrodes were patterned by photolithography and then deposited *via* thermal evaporation. A high-*k* Al_2_O_3_ gate oxide layer with a thickness of ~10 nm was formed using atomic layer deposition.

### Measurement

All electrical measurements were performed using a semiconductor analyzer (HP4155C, Agilent) at room temperature. The bending of the device was performed using custom-built bending stages.

### Simulation

The dimensional parameters are similar to those of the experimental device expect for the channel length of the access transistor, which is 1 *μ*m. In addition, the simulation models include the concentration-dependent Shockley-Read-Hall model, Lombardi mobility model, and Kane band-to-band tunneling model. Selberherr’s impact-ionization model is also considered for avalanche multiplication.

## Electronic supplementary material

Supplementary information
