# Supplementary material for: Memory characteristics of silicon nanowire transistors generated by weak impact ionization
Source: Sci Rep. 2017 Sep 29;7:12436. doi: 10.1038/s41598-017-12347-x (PMC5622113; doi:10.1038/s41598-017-12347-x)
Supplement: Supplementary file 1 — Supplementary information [file 41598_2017_12347_MOESM1_ESM.pdf]

Supplementary information for

Memory characteristics of silicon nanowire transistors generated by weak impact ionization

Doohyeok Lim, Minsuk Kim, Yoonjoong Kim, and Sangsig Kim*
Department of Electrical Engineering, Korea University, 146, Anam-ro, Sungbuk-gu, Seoul 02841, Korea

1. Latch-up/down phenomena generated by weak impact ionization of SiNW FET

The description of latch-up/down phenomena of the SiNW FET can be developed using an approach similar to the SOI-based device modeling.[S1] Similarly to the silicon-on-insulator (SOI)-based device, the expression of subthreshold swing (SS) in our SiNW FET is derived as:

$$\mathrm{SS} = \frac{kT}{q}\ln 10 \cdot n \qquad (1)$$

where $n = \frac{1+r}{1+r\frac{dV_{BS}}{dV_{GS}}}$ and $r = \frac{2\varepsilon_{Si}t_{ox}}{\varepsilon_{ox}t_{si}}$

In the SiNW FET, V_B is the potential at the body of silicon nanowire in the channel region, and V_{BS} is the difference between V_B and the voltage at source. In the latch-up/down phenomena of our device, the SS below thermal limit indicates that $dV_{BS}/dV_{GS} > 1$. To further understand these phenomena, an expression is derived to describe dV_{BS}/dV_{GS} by extending the analysis on SOI-based device to our device.[S1]

$$\frac{dV_{BS}}{dV_{GS}} \cong \frac{m}{n}\cdot\frac{1}{1+\frac{I_{gt}}{I_{gi}}} \propto \frac{m}{n}\frac{W}{L}exp\left[\frac{q}{kT}\left(\frac{V_{GS}}{n}-\frac{V_{BS}}{m}\right)\right]\cdot exp\left(-\frac{\beta_i l}{V_{DS}}\right) \qquad (2)$$

where I_{gt} is the off-state leakage current and I_{gi} is the impact ionization current.[S2] β_i is a constant and l is a structural parameter. m is the ideality factor of the junction between the channel region and the source region.

Compared to the fixed V_S, V_B continuously increases during the transition thanks to the increase of quasi-Fermi potential and the effects of the holes generated by weak impact ionization. Weak impact ionization is defined as the impact ionization, which only occurs during the transition and vanishes when the device completely turns on. Thus, the condition of latch occurrence with weak impact ionization is $I_{gi} = (M-1)I_{ch} \ll I_{ch}$, where I_{ch} is the channel current and M is the impact ionization multiplication factor.[S3] Moreover, the increase in dV_{BS}/dV_{GS} during the transition leads to the steep SS over the subthreshold region, *i.e.* latch-up/down phenomena.

2. Operating conditions for the SRAM array cell

The summary of operating conditions of the SRAM array cell is shown in table S1. The power supply (V_{DD}) should be applied for sustaining the state of the positive feedback loop. To write "1" data into the SRAM array cell, V_{WL1} is applied to generate the positive feedback loop while the access transistor controlled by V_{WL2} turns on, resulting in the latch-up state. For the erasing operation, V_{WL1} is applied to eliminate the positive feedback loop while the access transistor controlled by V_{WL2} turns on, leading to the latch-down state. The read operation is performed by sensing the difference in the bitline current between the latch-up state and the latch-down state. For the reading operation, the access transistor is kept completely *on* at V_{WL2} = 1 V, thereby sensing the state of the positive feedback loop. In addition, the access transistor is kept off for the hold operation. Thus, the hold current reaches the leakage current of the access transistor, which leads to reduction in power dissipation.

Operation	Pulse width	Bias voltage			
		V_{WL1}	V_{WL2}	V_{BL}	V_{DD}
Write "1" (programming)	5 ns	0 V	1 V	0 V	4.35 V
Write "0" (erasing)	5 ns	-4.6 V	1 V	0 V	4.35 V
Read	100 s	-2.3 V	1 V	0 V	4.35 V
Hold	1000 s	-2.3 V	0 V	0 V	4.35 V

Table S1. Operating conditions of the SRAM array cell.

3. The half-selected SRAM cell operations

Figure S1 shows the timing diagrams of an SRAM array cell including the half-selected cell operations at the writing pulse width of 5 ns. In the half-selected cell, the access transistor is kept off when WL1 signal is enabled. As shown in Figure S1, the WL1 signal does not disturb the data states when the access transistor is in off-state. Although the WL1 signal corresponds to the write “1” signal, the “0” state in the half-selected cell of the SiNW FET remains stable because the positive feedback loop cannot be formed without the current flowing. Also, the “1” state in the half-selected cell does not show the performance degradation when the WL1 signal reaches the write “0” signal. The generated positive feedback loop does not disappear without turning on the access transistor. Thus, the writing does not cause the error-writing operation in the half-selected cell when WL1 signal is enabled.

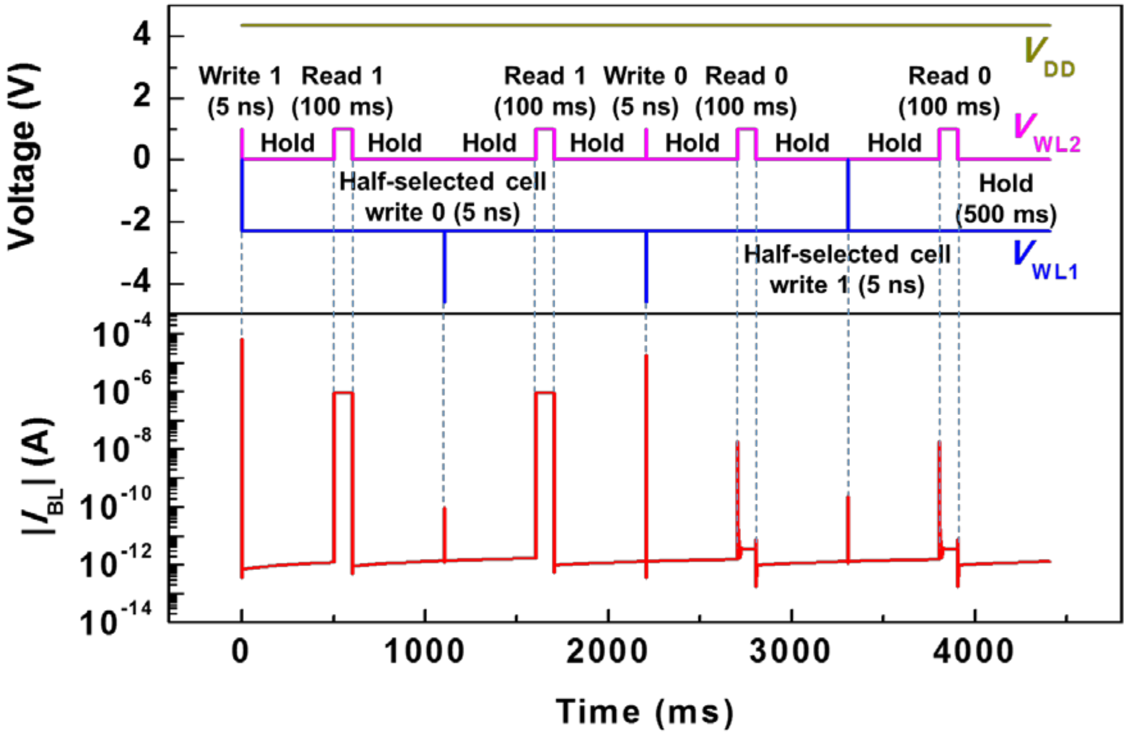

Figure S1. Timing diagrams of full operation.

4. Validation of the simulation results

Figure S2 shows the simulated transfer curve of the memory cell at V_{DS} = 4.35 V. In the calibration, the latch-up voltage ($V_{latch\text{-}up}$) and the latch-down voltage ($V_{latch\text{-}down}$) are the important factors to explain the positive feedback mechanism. Therefore, our simulation work focuses on calibrating the $V_{latch\text{-}up}$ and $V_{latch\text{-}down}$. In spite of the slight difference in the current level, the simulation results agree well with the experimental data. Moreover, the $V_{latch\text{-}up}$ and $V_{latch\text{-}down}$ in the simulation data correspond exactly to those in the experimental data as shown in Figure R2. Thus, the selected simulation models are proper to further investigate the memory characteristics.

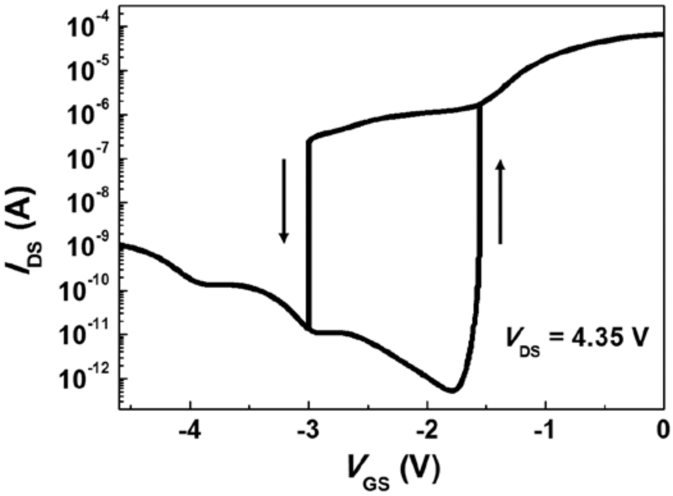

Figure S2. Simulated hysteresis characteristics in the I_{DS}-V_{GS} transfer curve at V_{DS} = 4.35 V.

References

(S1) Fossum, J. G. *et al*. Anomalous subthreshold current—Voltage characteristics of n-channel SOI MOSFET's, *IEEE Electron Device Lett.* **8**, 544-546 (1987).

(S2) Sze, S. M. & Ng, K. K. *Physics of semiconductor devices* (Wiley, 2006).

(S3) Fossum, J. G. & Lu, Z. Anomalous Floating-Body Effects in SOI MOSFETs: Low-Voltage CMOS? *IEEE International SOI Conference*, 1-2 (2011).
